# The multivariate analysis of variance as a powerful approach for circular data

**DOI:** 10.1186/s40462-022-00323-8

**Published:** 2022-04-27

**Authors:** Lukas Landler, Graeme D. Ruxton, E. Pascal Malkemper

**Affiliations:** 1 Institute of Zoology, University of Natural Resources and Life Sciences (BOKU), Gregor-Mendel-Straße 33, 1180 Vienna, Austria; 2 School of Biology, University of St Andrews, St Andrews, KY16 9TH UK; 3 Research Group Neurobiology of Magnetoreception, Max Planck Institute for Neurobiology of Behavior – caesar, Ludwig-Erhard-Allee 2, 53175 Bonn, Germany; 4 Department of Game Management and Wildlife Biology, Faculty of Forestry and Wood Sciences, Czech University of Life Sciences, 16521 Prague 6, Czech Republic

**Keywords:** MANOVA, Rayleigh test, Directional data, Orientation, Periodicity

## Abstract

**Background:**

A broad range of scientific studies involve taking measurements on a circular, rather than linear, scale (often variables related to times or orientations). For linear measures there is a well-established statistical toolkit based on linear modelling to explore the associations between this focal variable and potentially several explanatory factors and covariates. In contrast, statistical testing of circular data is much simpler, often involving either testing whether variation in the focal measurements departs from circular uniformity, or whether a single explanatory factor with two levels is supported.

**Methods:**

We use simulations and example data sets to investigate the usefulness of a MANOVA approach for circular data in comparison to commonly used statistical tests.

**Results:**

Here we demonstrate that a MANOVA approach based on the sines and cosines of the circular data is as powerful as the most-commonly used tests when testing deviation from a uniform distribution, while additionally offering extension to multi-factorial modelling that these conventional circular statistical tests do not.

**Conclusions:**

The herein presented MANOVA approach offers a substantial broadening of the scientific questions that can be addressed statistically using circular data.

**Supplementary Information:**

The online version contains supplementary material available at 10.1186/s40462-022-00323-8.

.

## Introduction

Some scales of measurement in science are inherently periodic rather than linear. Data on compass orientations, times of day and times of year are obvious examples of this. Such data are  often called circular, since it is easy to imagine the data being mapped onto the circumference of a circle. As soon as one considers that 355° is closer to 5° than it is to 340°, it is clear that such circular data needs different statistical treatment from linear variables (such as  mass and age), and a body of statistical theory has developed to allow investigation of circular data (for example: Batschelet [1], Fisher [2], Pewsey et al. [3] and Landler et al. [4]).

Rayleigh’s test on the null hypothesis that the population mean vector length is zero is often cited as the first statistical testing procedure designed for circular data. Since Lord Rayleigh [5] formulated his seminal Rayleigh test in the late nineteenth century, circular statistics as an academic discipline has steadily developed and expanded the range of possible approaches, nowadays including a myriad of test alternatives and software [6–8], second order analyses [9], as well as Akaike and Bayesian Information criterion methods [10–12]. All have the goal of helping researchers understand and correctly interpret data distributed on a circle. However, recently we confirmed that, despite all the developments, the Rayleigh test is still one of the most powerful tests for using a single sample to test the null hypothesis that the underlying population is uniformly distributed with no preferred direction [4]. In contrast to some alternative tests, the Rayleigh test also controls type I error rates for aggregated data (e.g. if directions are only recorded to the nearest degree, or even ten degrees) and can be used to detect a variety of deviations from uniformity, with the exception of multimodal symmetrical distributions [13, 14].

One feature all commonly-used tests for circular statistics based on null-hypothesis testing have in common is the inability to use multiple covariates and/or multiple explanatory factors if the circular variable is the response. That is, investigation is limited to a single independent variable (although this variable can be discrete, continuous and circular, or continuous and linear). This is in stark contrast to analyses of linear dependent variables, where multiple independent variables are analyzed routinely within one testing procedure.

In a recent analysis we compared a suite of available tests designed to compare two samples to test the null hypothesis that they come from the same underlying population and found that also in this testing situation a very common test, the Watson U^2^ test [15], showed superior power to many other standard options [16]. Within this study, we also experimented with a new approach, which exploits a well-known procedure to linearize circular data by using sines and cosines of the angles (see for example Pewsey et al. [3]). In order to use two linear factors (the sines and cosines of the recorded angles) as dependent variables, we needed to switch from univariate linear models to a multivariate analysis of variance. This approach allows the use of multiple response variables [17]. In our study we used MANOVA to test for a difference of two circular distributions by using the sample id as a factor with two levels (see Pail et al. [18] for one earlier study that used a MANOVA to analyze effects of a linear variable on a directional response, and Sect. 7.4 of Mardia & Jupp [19] for methods to deal with more than two distributions). Perhaps surprisingly, this novel approach offered very similar performance to the best-established tests in this simple testing situation, for instance, the Watson U^2^ test [16]. It is important to add that the sine and cosine of an angle (theta), while derived from the same number, are orthogonal to each other by definition. This fact might add to the power of the MANOVA approach.

Although our previous investigation was restricted to a single two-level explanatory factor, it is clear that the MANOVA approach allows for the use of more than two levels per factor, multiple grouping factors, as well as linear covariates [20–22]. However, it is less clear how this would relate to circular distributions, or in other words, how p values obtained in such more-complex analyses would relate to specific null hypotheses.

While we have already shown that differences between two populations can be reliably tested, we wanted to also use this approach to test for significantly clustered (i.e., non-random) unimodal distributions based on just a single sample of data. Testing the null-hypothesis that a single sample of data comes from an underlying uniform distribution is very likely the most commonly applied procedure in circular statistics. We hypothesized that the intercept of the null model without any explanatory variables could be used to derive p values related to significant clustering. As such approach would be novel to circular statistics, we tested this hypothesis and compared this MANOVA intercept approach with the two most powerful tests for unimodal deviations from uniformity in a single sample, the Rayleigh and Hermans–Rasson tests [4, 23]. We then simulated some example situations with known underlying distributions to further explore the MANOVA approach’s general usefulness in situations including grouping and linear factors.

This paper explores the use of MANOVA applied to circular response variables, and provides advice for people interested in using this for their own research. We show that the intercept of the MANOVA models is a reliable proxy of unimodal clustering and that grouping factors as well as linear covariates can be accommodated in the very flexible approach, which considerably enhances model performance and interpretation of circular data.

## Methods

### Statistical tests used

All analyses were performed in the statistical software R [24], R code used for simulations and R output can be found in the Additional file 1 (the R code and example data can also be found on github: https://github.com/Malkemperlab/Circular-MANOVA). For all analyses we performed the MANOVA approaches using the summary function for the “manova” function in base R. We exploited a common transformation of circular variables into two orthogonal linear variables for the response matrix. This was achieved by using sine and cosine of the angle (in radians). This approach has previously been used to perform linear models with angles as independent variables (see [3]). If the angles are viewed as vectors on a unit circle, the sine and cosine of the angle represent the x and y component of such a vector. However, this view might only be valid for the case of a MANOVA model without any independent variables (intercept only). In such a special case, a significant p value is expected to indicate that the (vector) distribution differs significantly from the center of such unit circle (0,0). Following this logic, independent factors can then change the weight (length) of the vectors (linear variables) or group the vectors and allow testing for differences between the groups (grouping factors).

In our power analysis the independent variables were either absent (intercept-only approach) or different for each of the analyses. The intercept-only approach used no response variables, therefore, only a single p value was reported. In the linear MANOVA approach, a linear variable was added, therefore reporting the p value of the intercept and the covariate. In the grouped MANOVA approach a grouping factor was added, therefore, generating p values for both the intercept and the grouping factor. In the last variant of MANOVA model explored, linear and grouping factors were combined in one MANOVA analysis, allowing the analysis of significant intercept, linear and grouping effects. Throughout, we compared two MANOVA approaches, using both conventional theory and numerical simulation to evaluate p values. For the simulation-based MANOVA approaches, the obtained p values were adjusted by comparing them to 9999 iterations of the same analysis using samples from a uniform random distribution. The Rayleigh test was calculated using the function rayleigh.test of the package *circular* [8]. The Hermans-Rasson (HR) test was performed using the R package *CircMLE* [10] and function HermansRasson2T [23].

### Simulation tests

All tests described above were applied to the following samples, which were drawn from the given distribution (9999 iterations), power was defined as the proportion of significant test results (p< 0.05). For all simulations circular distributions were generated using the function rcircmix from the package *NPcirc* [25].

First, in order to test type I error rate, we simulated samples from circular uniform distributions (type “unif” in rcircmix, sample sizes: 5, 10, 15, 25, 50 and 100), from a continuous as well as binned distribution (with data aggregated into 10° bins around the circle, for all binned data the minimum sample size used was 10). The binning was done to test for sensitivity to such rounding of measured data, which can cause issues with some circular tests [14].

In a next step, we tested samples drawn from a unimodal von Mises (type “vm”, kappa = 1, mean direction: 0, sample sizes: 5, 10, 15, 25, 50 and 100) and wrapped skew normal (type “wsn”, dispersion parameter = 2, skewness: 30, location parameter: 0, sample sizes: 5, 10, 15, 25, 50 and 100) distribution. We then simulated samples from both a symmetrical (mean directions: 0° and 180°) and an asymmetrical (mean directions: 0° and 240°) bimodal von Mises distribution (type “vm”, kappa = 1, sample sizes: 10,20,30,50,100,200). To further extend this power comparison of basic distributions, we used samples from symmetrical (mean directions: 0°, 120° and 240°) and asymmetrical (mean directions: 0°, 120° and 270°) trimodal von Mises distributions (type “vm”, kappa = 1, sample sizes: 15, 30, 45, 75, 150, 300).

In order to gain insights into the usefulness of the MANOVA approach for comparisons we simulated data for a hypothetical scenario. In this scenario, we work on an animal population where we know the age and sex (called group 1 and group 2 herein) of the tested individuals. The assumption is that the homeward orientation after displacement becomes more clustered with age (which in this example ranges from 1 year to 5 years) and that group 1 is either less clustered or directionally differently oriented than group 2. To keep the presentation consistent, data were plotted according to the sample size of the potential treatment combination. Two groups and five ages resulted in 10 treatment combinations, hence the minimum sample size was 10.

### Real data examples

In order to test the presented approach on real data, we used three publicly available data sets from studies on animal behavior. The first one is a data set from Gagliardo et al. [26] embedded in the R package *circular*. In this study a translocation experiment was performed on pigeons, with three groups: control, sectioned olfactory nerve and section of the ophthalmic branch of the trigeminal nerve. The expectations were a difference between groups, an overall orientation towards the home direction, and no directional clustering in the group with the sectioned olfactory nerve.

The second data set was from Lindecke et al. [27] involving migratory bats (*Pipistrella pygmaeus*). The expectation was a difference between two treatment groups (with a 180° switch in preferred direction) and an effect of age on the treatment effect (interaction between age and treatment). For our analysis we started with a full model including the factors age, sex, treatment, temperature and wind speed. Then we used our self-written AIC based model selection function (see Additional file 1). This function first orders the independent variables based on their Eta-squared values from the full model (using the function *eta_squared* from the package *effectsize* [28] on both underlying ANOVAs, i.e., for each response variable). The first *n*-variables are then used in an AIC based model comparison (*n* can be specified in the function input - in the case of the bat examples no variable was discarded at this step). For the AIC based model comparison all possible variable combinations are used in a MANOVA model and ranked according to their AIC values (mean AIC from both underlying ANOVAs, i.e., for each of the two response variables).

The third data set is from Obleser et al. [29], where the flight direction of deer was investigated. This data set included numerous potential explanatory variables, i.e. hour of the day, temperature, light intensity, wind speed, wind direction, sun direction, hide direction, previous alignment, distance of the hide, group size, sex, age, observer direction and vegetation height, which were included in a full model. In a further step we used our AIC based model selection function to reduce the model (the first 10 independent variables were used in the first selection step to allow fast calculation, see above for explanation). The original study observed a north-south flight direction of roe deer; thus the dependent variable was hypothesized to be axial. We, therefore, doubled the angles and reduced to modulo 360° (= 2*pi in radians) for our analysis [1]. All independent variables were added as axial as well as non-transformed directions. Hence this data set represented a complex system of multiple variables. In the published paper this was overcome by performing several different graphical and statistical analyses, in our approach we tested all the underlying hypotheses in one single model.

## Results

### Type I error

**Fig. 1:**
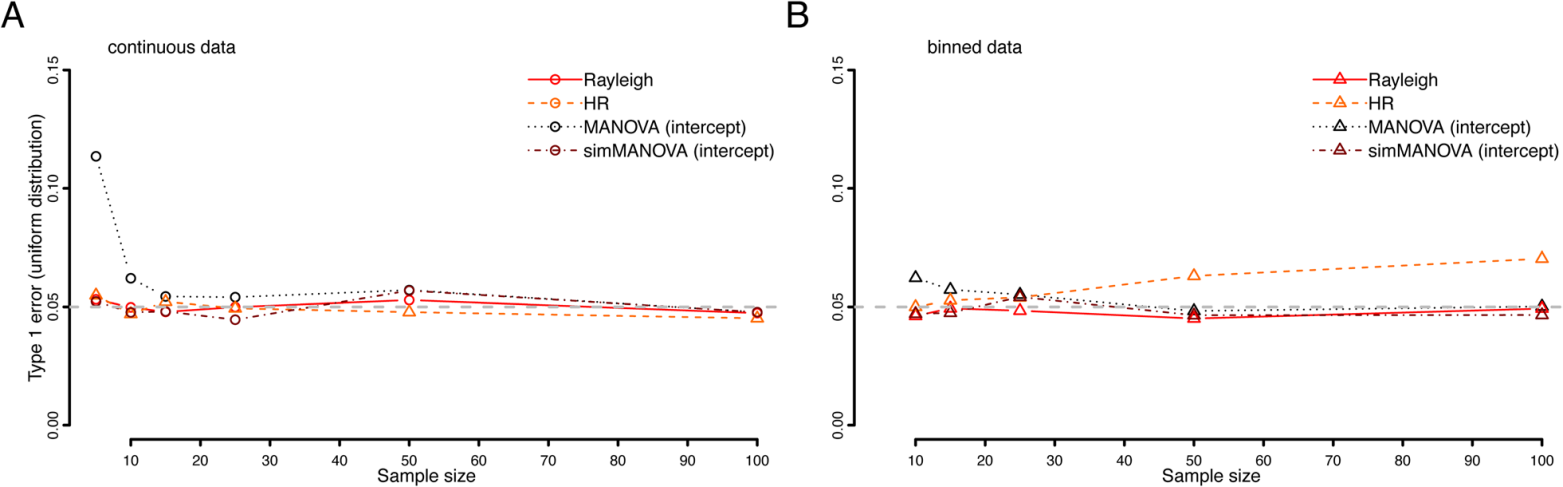
Type I error rates at different sample sizes of the four statistical tests used for continuous data (**a**) and binned data (**b**)

Type I error was slightly above 0.05 at very low sample sizes for the MANOVA approach but kept expected alpha values at sample sizes of at least 10–15 (Fig. 1). The simulation MANOVA approach controlled type I error throughout all sample sizes. The HR test showed slightly increasing type I error rates with increasing sample sizes for binned data (10° bins), as demonstrated earlier [14].

### Power analysis—basic distributions

**Fig. 2:**
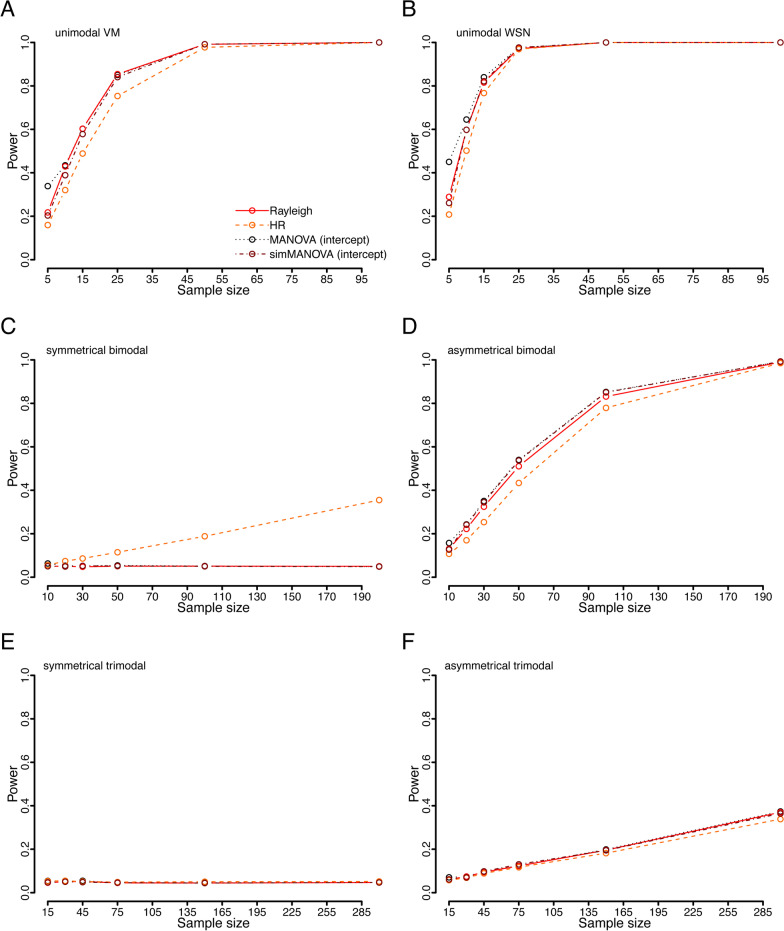
Power of the four statistical tests in situations with different underlying distributions: unimodal von Mises (**a**), unimodal wrapped skew normal (**b**), symmetrical bimodal von Mises (**c**), asymmetrical bimodal von Mises (**d**), symmetrical trimodal von Mises (E), asymmetrical trimodal von Mises (**f**)

For unimodal distributions the MANOVA approach and the Rayleigh test performed equally well, with the HR test showing just slightly lower power (Fig. 2a, b). The standard MANOVA approach showed artificially increased power levels at a sample size of 5, due to increased type I error rate at such small sample sizes. This was controlled for by the simulation MANOVA approach. When evaluating symmetrical bimodal distributions (Fig. 2c), the HR test was the only test with useful power to detect clustering, however, for asymmetrical bimodal distributions (Fig. 2d), the results were similar to the unimodal distribution, with the HR test performing slightly worse than both the other tests. None of the tests were able to detect clustering in symmetrical trimodal distributions (Fig. 2e) over the range of sample size used (15–300). All of the tests had very similar (low) power to detect clustering of asymmetrical trimodal distributions (Fig. 2f). Taken all the analyses together, for the standard distributions, the power of the MANOVA approach was almost identical to the very powerful Rayleigh test, and only shared the same weakness of low power with symmetrical distributions, which traditionally can be overcome by simple data transformation [1].

### Power analysis—hypothetical examples

**Fig. 3:**
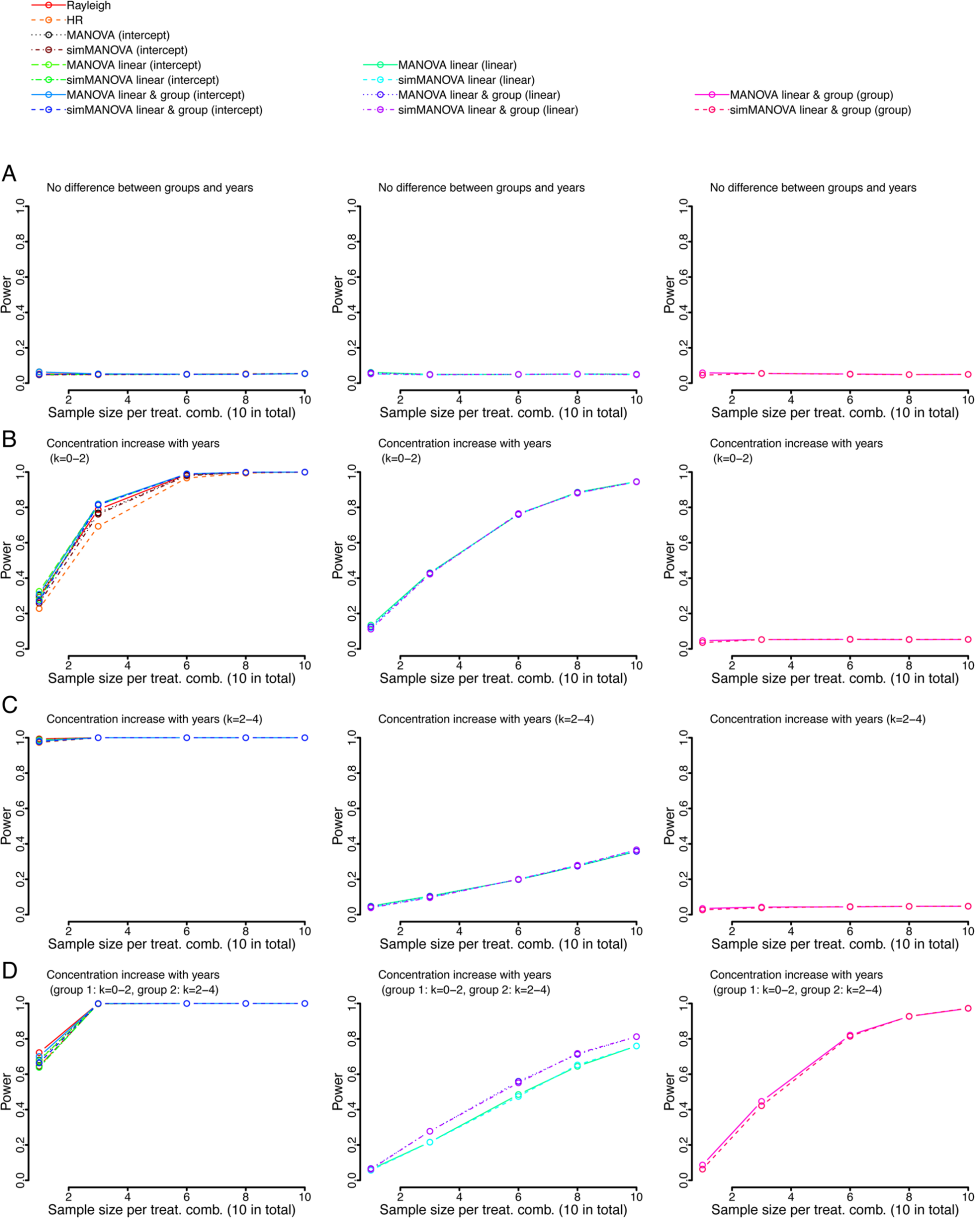
Power of several hypothetical examples for the proposed approach. In a hypothetical 5-year study (linear variable) of a population composed of two groups (grouping variable) the orientation performance of subjects is measured. P values for the MANOVA intercepts as well as Rayleigh and HR test are shown in the left panel, p values for the linear variables in the center and grouping variables in the right panel. In the case of randomized data (**a**), all p values of all measured tests remained at nominal levels. In all following distributions the clustering of orientations continuously increased each year, however, mean orientation was identical between all groups. First, we only changed the yearly increase from 0–2 (**b**) to 2–4 (**c**). In a second step, we used different yearly clustering increases for group 1 and group 2 (**d**)

**Fig. 4:**
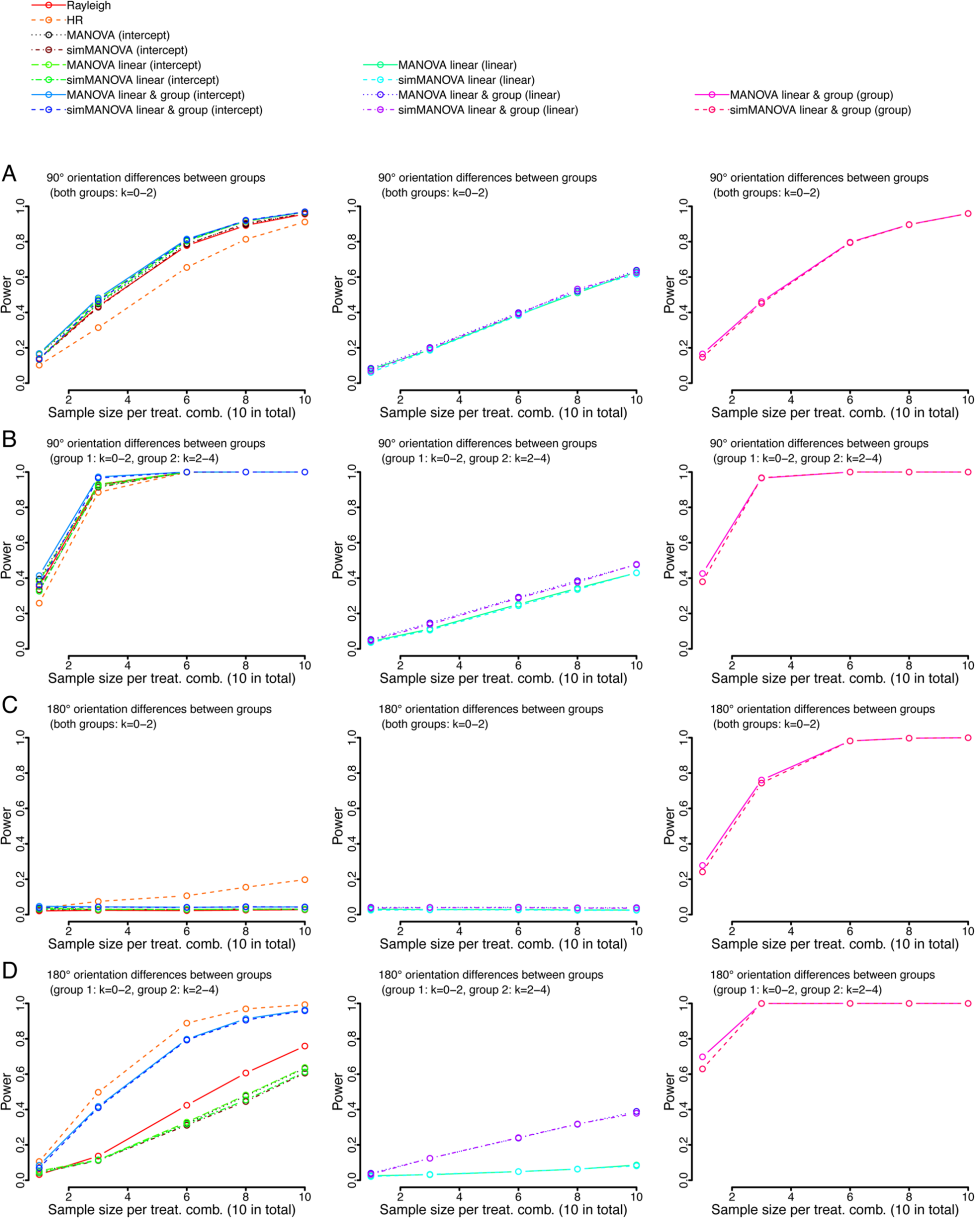
Continuation of our hypothetical example, analyzing the usefulness of the MANOVA approach for biological data. In addition to the yearly increase of clustering, we now manipulated the orientation direction for each group, using either a 90° (**a**, **b**) or a 180° (**c**, **d**) difference. This was then either combined with a group difference in yearly clustering increase (**b**, **d**), or not (**a**, **c**). The left panel shows the power for the intercept of MANOVA approaches as well as Rayleigh and HR test, the center panel shows the power of detecting the linear effect, the right panel shows power for detecting group differences

All tests controlled type I error when all treatment combinations were sampled from the same distributions (Fig. 3a). The overall power to detect orientation non-uniformity was similar between tests when the mean direction was the same for all distributions, however, the MANOVA approach in addition appropriately identified a linear and/or grouping effect (Fig. 3b–d). In the case of differing mean directions between groups, the MANOVA approaches performed very well in detecting deviations from uniformity (Fig. 4). The exception was the case of two groups with mean orientations 180° apart, in this special case only the HR test showed moderate power to detect non-random orientation (Fig. 4c). However, the MANOVA approach showed good power to detect differences between groups, and hence an effect on orientation. The MANOVA approach performed almost as well as the HR test when there was a linear effect (different between groups) added to the two groups with opposite orientations (Fig. 4d).

### Real data examples

The MANOVA performed on the pigeon data showed a highly significant treatment effect and an overall significant non-uniform orientation (intercept) (Table 1), which is in line with the conclusions made in the original study [26].

**Table 1:** MANOVA table for the pigeon example, showing the significant overall orientation (intercept) and treatment effect

Factor	Pillai	approx. F	p
Intercept	0.63	89.50	< 0.01
Treatment	0.30	9.31	< 0.01

For the bat example, the MANOVA analysis elegantly combined all possible separate analyses in one model and showed that the treatment as well as age-by-treatment interaction were both significant (Table 2). Hence, the treatment effect changes with age of the bat. This corroborates (in a much more compact way) the results already presented in the original study by Lindecke et al. [27]. The overall orientation (intercept) was not significant, which is expected as the treatment groups showed opposite (overall axial) orientation.

**Table 2:** MANOVA table for the bat example, showing the significant treatment effect, as well as interaction with age

Factor	Pillai	approx. F	p
Intercept	0.02	0.52	0.598
Age	0.05	1.23	0.302
Treatment	0.21	5.82	< 0.01
Age by treatment	0.15	3.82	0.029

For the deer example, our approach showed the potential of the MANOVA approach to handle multiple potential explanatory variables. While in the original study numerous separate analyses were made and effect strengths compared [28], we can show in our rudimentary model selection approach that the axis of the hide as well as the previous alignment of the deer axis influenced the flight direction the most, i.e. were retained in the final model and showed a highly significant p value (Table 3). This supports the idea brought forward in the paper by Obleser et al. [29], that animals tend to align along the magnetic north-south axis, and (even more so) use the same axis as their preferred flight direction.

**Table 3:** MANOVA table of the selected model, after eliminating non-significant factors, for the deer example, showing significant effects of the previous axial alignment as well as the hide axis—expressed as cosine and sine of the axial directions in radians. In addition, sun axis, wind speed, wind direction and temperature showed marginal significant/trending effects

Row	Pillai	approx. F	p
Intercept	0.07	6.56	< 0.01
Cosine of previous alignment	0.09	8.03	< 0.01
Cosine of hide axis	0.08	7.42	< 0.01
Sine of hide axis	0.08	7.11	< 0.01
Sine of sun axis	0.03	2.87	0.06
Wind speed	0.03	2.67	0.072
Cosine of wind direction	0.03	2.32	0.101
Temperature	0.04	3.17	0.044

## Discussion

Our previous published work demonstrated that MANOVA is as powerful as the available conventional tests to investigate whether two samples come from the same underlying distribution. Here we have demonstrated that the effectiveness of this approach extends much more widely. Firstly, our analysis shows that the MANOVA approach for circular data is as powerful to determine significant departure from uniformity in a single sample of data as the popular and effective Rayleigh test. In addition, adding linear and grouping variables can vastly improve the power of the MANOVA, but also enhance the range of hypothesis testing. If one suspected that a certain linear variable or a grouping factor might be responsible for a change in orientation this can be tested explicitly without applying multiple tests (i.e., avoiding the issue of multiple hypothesis testing and inflating the type I error). There are no restrictions on the type of distributions to be used for this approach.

A potential weakness of the conventional MANOVA approach (where the p values are obtained by theoretical approaches) is the increased type I error rate for very low sample sizes (i.e., over all of n < 15). However, in such cases the p value can be calculated by Monte Carlo simulations, in this case it retains power levels and controls alpha inflation. We provide R code to perform this test in Additional file 1. If symmetrical multimodal orientations can be expected in a diversity of real-world situations generating circular data, the data can be transformed prior to analysis by MANOVA (see our deer example for axial data). However, if cofactors (or groupings) are not needed, and the type of modality is not known, one might prefer to use the HR test, which is more powerful than most other available options in such a situation.

In contrast to other more sophisticated circular models, e.g. the Bayesian GLM [11], our approach provides insight into the circular response variable and its deviation from uniformity for single samples, in addition to testing for the significant contributions of groups and linear factors.

In principle, MANOVAs might be used to incorporate random effects as well, e.g., in a repeated-measures MANOVA. However, such approach will require validation for the intercept approach described here, this potentially opens the possibility to perform experiments and accompanying analyses without any of the limitations previously experienced in circular statistics. Thus, the MANOVA approach demonstrated here, in combination with recent developments in Bayesian approaches, offers a substantial broadening of the scientific questions that can be addressed statistically with circular data. The fact that it also performs well when testing very simple hypotheses that are currently evaluated by a collection of specialist tests, should make its wider adoption all the more attractive.

## Conclusions

We show that the herein presented MANOVA approach has the potential to extend the range of scientific questions substantially that can be tested statistically using circular data. In addition, using such approach would lead to more powerful analyses than currently possible and paves the way to make the linear statistical toolbox available for circular data.

## Supplementary Information

**Additional file 1**. R-code and example data to perform the statistical tests described in the manuscript.

## Data Availability

Data and code are available in the Additional file 1.
